# Comparative analysis of the dust retention capacity and leaf microstructure of 11 *Sophora japonica* clones

**DOI:** 10.1371/journal.pone.0254627

**Published:** 2021-09-07

**Authors:** Jie Yu, Li-Ren Xu, Chong Liu, Yong-Tan Li, Xin-Bo Pang, Zhao-Hua Liu, Min-Sheng Yang, Yan-Hui Li

**Affiliations:** 1 Hebei Key Laboratory for Tree Genetic Resources and Forest Protection, Hebei, Baoding 071000, China; 2 Institute of Forest Biotechnology, Forestry College, Agricultural University of Hebei, Baoding 071000, China; 3 Forest City Construction Technology Innovation Center of Hebei, Shijiazhuang 050000, China; 4 College of Landscape Architecture and Tourism, Agricultural University of Hebei, Baoding 071000, China; 5 Hongyashan State Owned Forest Farm, Hebei, Baoding 071000, China; Universidade Federal de Alfenas, BRAZIL

## Abstract

We used fresh leaves of *Sophora japonica* L. variety ‘Qingyun 1’ (A0) and 10 superior clones of the same species (A1–A10) to explore leaf morphological characteristics and total particle retention per unit leaf area under natural and artificial simulated dust deposition treatments. Our objectives were to explore the relationship between the two methods and to assess particle size distribution, X-ray fluorescence (XRF) heavy metal content, and scanning electron and atomic force microscopy (SEM and AFM) characteristics of leaf surface microstructure. Using the membership function method, we evaluated the dust retention capacity of each clone based on the mean degree of membership of its dust retention index. Using correlation analysis, we selected leaf morphological and SEM and AFM indices related significantly to dust retention capacity. *Sophora japonica* showed excellent overall dust retention capacity, although this capacity differed among clones. A5 had the strongest overall retention capacity, A2 had the strongest retention capacity for PM_2.5_, A9 had the strongest retention capacity for PM_2.5–10_, A0 had the strongest retention capacity for PM_>10_, and A2 had the strongest specific surface area (SSA) and heavy metal adsorption capacity. Overall, A1 had the strongest comprehensive dust retention ability, A5 was intermediate, and A7 had the weakest capacity. Certain leaf morphological and SEM and AFM characteristic indices correlated significantly with the dust retention capacity.

## 1. Introduction

Atmospheric particulate matter has become the primary pollutant affecting air quality in China in recent years due to rapid industrialization and urbanization. Atmospheric particulate matter pollution reduces visibility and triggers a variety of environmental problems, such as haze. Based on aerodynamic diameter (D), atmospheric particles may be divided into total suspended particles (PM_100_; D ≤ 100 μm), respirable particles (PM_10;_ D ≤ 10 μm), and fine particles (PM_2.5_; D < 2.5 μm). Particles smaller than PM_10_ may be inhaled into the human body, causing serious health impacts [1,2]. Many studies have shown that plants, with their unique leaf surface structure, can effectively block and absorb atmospheric particulate pollutants, and have a certain capacity to adsorb harmful heavy metals [3–5].

To date, studies of the dust retention capacity of landscaping trees have focused mainly on species comparisons, primarily using single dust retention indices. No study has examined the dust retention capacity of different clones of the same species [6–9]. In addition, artificial dust fall has been used to simulate dust deposition under natural conditions, but the correlation between natural and artificial conditions requires further study [10]. Scanning electron microscopy (SEM) is an observational method that falls between transmission electron microscopy and optical microscopy. Microscopic variation in leaf surface structure can be examined in detail using SEM [11,12]. Atomic force microscopy (AFM) is used to assess the surface structure and properties of matter by detecting very weak interatomic interaction between the surface of the sample and a miniature force-sensitive element. AFM can be used to assess the roughness of plant leaves and the characteristics of leaf surface structure [13,14]. At present, the combined use of SEM and AFM is widespread in materials analysis, chemistry, and other fields [15–18], but its use to study plants has not been reported. X-ray fluorescence (XRF) is a spectral analysis technique falling between the atomic emission and atomic absorption spectra. By measuring the wavelength and intensity of a series of XRF lines, the type and amounts of elements can be determined. XRF has many advantages over the traditional atomic absorption method, such as the ability to use smaller samples, the shorter analysis time, the ability to examine a wide range of analytical elements, its high accuracy and lesser sensitivity to experimental errors, and its low risk coefficients. It has been used widely in biology, medicine, criminal investigations, and other fields [19–21], but it has not been applied in forestry or horticulture.

*Sophora japonica* L. is a perennial deciduous tree in the genus *Sophora* and the family Leguminosae. It is a common ornamental tree species in China, often used as a shade or street tree. The species can reach 25 m in height and exhibits strong sprouting ability and fast growth. It plays important roles in water and soil conservation, wind mitigation, and soil stabilization. *S*. *japonica* has traditionally been used to improve air quality, but its anti-pollution mechanisms are not well understood [22,23]. We analyzed the leaf phenotypic traits of 11 high-quality clones of *S*. *japonica*, and compared the dust retention ability of leaves under natural and artificial simulated conditions. We used the analysis of retained particles to create a particle size distribution diagram. Furthermore, we used XRF technology to qualitatively and quantitatively analyze heavy metals in retained particulates, using the membership function method to comprehensively evaluate the dust retention ability of clones by integrating all dust retention indices. Using the SEM and AFM cross-linking method, we assessed the microscopic characteristics of the leaf surface in detail, and analyzed the correlation with each dust retention index to identify morphological and surface microstructural characteristics that lead to differences in dust retention capacity. Our objective was to provide a practical reference for use in breeding new *S*. *japonica* varieties, horticultural applications of the species, and future studies on related topics.

## 2. Materials and methods

### 2.1 Materials

The test site was at the Hongyashan State-owned Forest Farm (39° 35′ 99″ N, 115° 56′ 36″ E) in Baoding City, Hebei Province, in the temperate continental monsoon climate zone. According to the 2019 environmental quality bulletin of Baoding City, the first-class air quality standard is attained in the city on only 30 days of the year, and the average annual concentration of PM_2.5_ is 58 μg·m^–3^. Air pollution has seriously affected human health

Experimental materials included the ‘Qingyun1’ variety of S. japonica (A0) [24], selected by the Hebei Academy of Forestry Sciences, as well as 10 clones with straight trunk, robust growth and relatively uniform height were selected as materials (A1–A10). Materials were selected in 2016 from the seed forest at the forest farm. In March 2019, we established an experimental forest of clones using a complete random block design comprising three groups, with 11 plots per group and 6 replicates per plot. Seedlings of all clones were propagated uniformly as 2-year-old grafts, and the row spacing was 3 × 4 m.the clone information of each trial was shown in Table 1.

**Table 1:** Clones tested.

Sample number	Tree height/m	Breast diameter/cm	Ground diameter/cm	Crown width/m	
				East and west	North and South
A0	5.04±0.46	5.06±0.90	7.32±1.23	1.55±0.64	1.44±0.65
A1	5.43±0.47	5.10±0.89	7.30±1.30	1.28±0.456	1.46±0.63
A2	5.14±0.32	5.03±0.82	7.40±1.03	1.71±0.84	1.44±0.73
A3	5.23±0.53	4.98±0.91	7.10±1.55	1.58±0.53	1.61±0.54
A4	5.63±0.48	4.70±0.42	7.05±0.74	1.13±0.37	1.30±0.53
A5	5.34±0.48	4.88±0.73	6.90±1.10	1.40±0.38	1.37±0.42
A6	4.88±0.38	4.24±0.55	6.71±1.23	0.79±0.32	0.74±0.35
A7	5.46±0.48	5.16±0.51	7.65±1.06	1.31±0.35	1.37±0.32
A8	5.70±0.58	5.19±0.97	7.89±1.53	1.68±0.79	1.83±0.81
A9	5.44±0.36	5.25±0.69	7.45±1.23	2.15±0.60	1.91±0.47
A10	5.21±0.58	4.39±0.63	6.28±1.01	1.09±0.42	1.19±0.50

### 2.2 Methods

#### 2.2.1 Morphological indices

On August 21, 2020, six vigorous, pest-free plants of each clone were selected. Approximately 50 g of healthy leaves was collected from the upper, middle, and lower canopy layers, from east-, south-, west-, and north-facing branches. The leaves were scanned as JPEG files (LiDE300; Canon, Tokyo, Japan), which were imported into Adobe Photoshop CS5 (Adobe, San Jose, CA, USA) and Lamina. Leaf length and width, petiole length, and leaflet length, width, perimeter, and area were measured.

#### 2.2.2 Measurement of retained particles in leaves

*2*.*2*.*2*.*1 Particle retention under artificial conditions*. On September 15, 2020, 1 day after a large precipitation event, we selected and sampled leaves as described in Section 2.2.1. The samples were sealed in self-sealing PE bags and brought to the main laboratory of the Forest Germplasm Resources and Forest Protection of Hebei Province for artificial, simulated dust exposure, following the methods of Guo *et al*. [10]. A large amount of road dust mixed with loess was used as an artificial dust source. It was placed into a 140-mesh sieve (D = 106 μm) located adjacent to the upper 10 cm of the leaf and shaken at a consistent rate to ensure even sprinkling of dust on leaf surfaces, until dust began to slide off the leaves. The total mass of dust retained on the leaf surface was then measured. This procedure was replicated three times for each clone.

Following the method described by Hong *et al*. [25], after the collected leaves are fully soaked in deionized water, they are washed with distilled water of an ultrasonic cleaner. The resulting suspension (M_T_) was stirred for 5–7 min using a constant temperature magnetic agitator to disperse the particles uniformly. Approximately 30–50 mL of the suspension was then placed into a Petri dish, and the quality of a portion of the suspension was assessed (M_p_). The Petri dish was placed in an oven at 60°C and dried to a constant mass, and the mass of the particles (m_p_) was obtained by weighing. The total mass of particulate matter retained on the leaf surface (M) was calculated as follows:

$$M=m_{p}\cdot M_{T}/M_{P}$$

*2*.*2*.*2*.*2 Particle retention under natural conditions*. On September 22, 2020, following a 7-day period without rainfall and with wind speeds below level 5, we sampled leaves using the method described in Section 2.2.2.1. The leaves were cleaned in an ultrasonic cleaner, and particle quality was assessed as described above.

*2*.*2*.*2*.*3 Leaf area*. Leaf area was calculated using the method described in Section 2.2.1.

#### 2.2.3 Particle size distribution

We extracted 30 mL of the suspensions (see Section 2.2.2.2) from each sample into 50-mL PE centrifuge tubes. Following ultrasonic shock, particle size was analyzed using a Mastersizer2000 laser particle size analyzer (Malvern Panalytical, Malvern, UK). Each treatment was replicated three times.

#### 2.2.4 XRF test for heavy metals

We collected particulate matter samples after drying as described in sections 2.2.2.1 and 2.2.2.2, and mixed the samples according to clone number. The XRF of each dust sample was tested using an X-MET7500 fluorescence spectrometer (Oxford Instruments, Abingdon, UK). The procedure was replicated three times for each clone.

#### 2.2.5 Leaf surface characterization

After suctioning of the surface moisture from washed leaves (see Section 2.2.2.2), holes were punched on either of the midveins using a 1-mm round hole punch. Following the method of Zhang *et al*. [26], the punched discs were pre-treated, freeze dried, gold plated, and then observed and photographed using an SU8100 cold field scanning electron microscope (Hitachi, Tokyo, Japan).

The remaining discs were scanned at room temperature in non-contact mode, following the method of Li *et al*. [13], using an atomic force microscope (SR13800-SRA-400). All AFM images were taken in height mode without processing.

#### 2.2.6 Data analysis

Excel 2016, SPSS 25.0, and DPS 7.05 were used for statistical analyses and the calculation of distance matrices. MEGA 7 and GraphPad Prism 8.0 were used to visualize the results.Following the method of Guo *et al*. [27], and using the membership function method, we calculated the degree of membership of each dust retention index as follows:

$$U_{ij}=(X_{ij}-X_{jmin})/(X_{jmax}-X_{jmin}),$$

where *U*_*ij*_ is the membership function value of sample *j*, index number *i*, *X*_*ij*_ is the measured value of sample *j*, index number *i*, *X*_*jmin*_ is the minimum value among all sample *j* indicators, and *X*_*jmax*_ is the maximum value among all sample *j* indicators. The average degree of membership for each clone was used as a comprehensive standard for evaluation of the dust retention capacity.

## 3. Results and analysis

### 3.1 Dust retention capacity of different clones

#### 3.1.1 Particle retention under natural and artificial conditions

**Fig 1:**
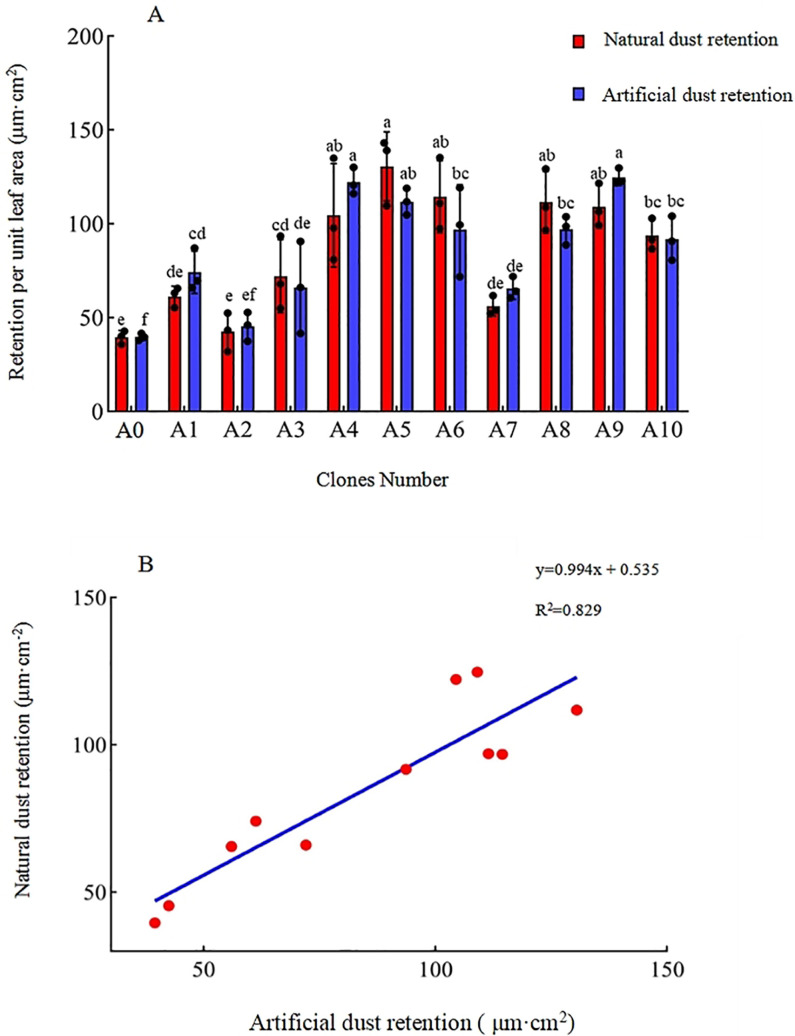
Retention capacity of total particulate matter in leaves of different clones of *Sophora japonica* L. Data in 2A are means ± standard deviation. Different lowercase letters indicate significant differences among different clones using the same determination method (*p* < 0.05).

The dust retention capacity per unit leaf area reflects the overall capacity of leaves to retain airborne particulate matter. Ranges of variation in unit leaf area retention among the clones under natural and artificial conditions were 39.526–130.580 and 39.678–124.758 μg·cm^2^, respectively (Fig 1A), with no difference between methods.

Under natural conditions, A5 exhibited the greatest retention of total particulate matter per unit leaf area, 17.10–230.36% greater than that of other clones. Multiple comparisons indicated that aside from A4, A6, A8, and A9 and no significant difference compared with A5, the other samples were significantly lower. Under artificial conditions, A9 exhibited the greatest retention of total particulate matter per unit leaf area, 2.06–214.43% greater than that of other clones. Multiple comparisons indicated that all clones other than A4, A5 and A9 exhibited significantly lesser retention capacities. Under both treatments, A4, A5, and A9 exhibited greater capacity to retain total atmospheric particulate matter.

The coefficient of variation (COV) is an indicator of data stability. The average COVs under natural and artificial conditions were 15.37% and 13.08%, respectively. These COV values < 20% indicate that the data obtained using the two methods are stable and reliable. The results obtained under artificial conditions were more stable than those obtained under natural conditions. To explore the correlation between methods, we fitted a linear regression equation using natural conditions as the dependent variable and artificial conditions as the independent variable. The equation was *y* = 0.994*x* + 0.535, with an *R*^2^ value of 0.829, indicating a strong linear relationship between methods (Fig 1B). We also detected a significant positive correlation (correlation coefficient = 0.9103, *p* < 0.01), implying that the results obtained under artificial, simulated conditions are comparable to those obtained under natural conditions. In future studies, this method may be used as an alternative in case of uncontrollable external factors, such as extreme weather.

#### 3.1.2 Retained particle size

**Fig 2:**
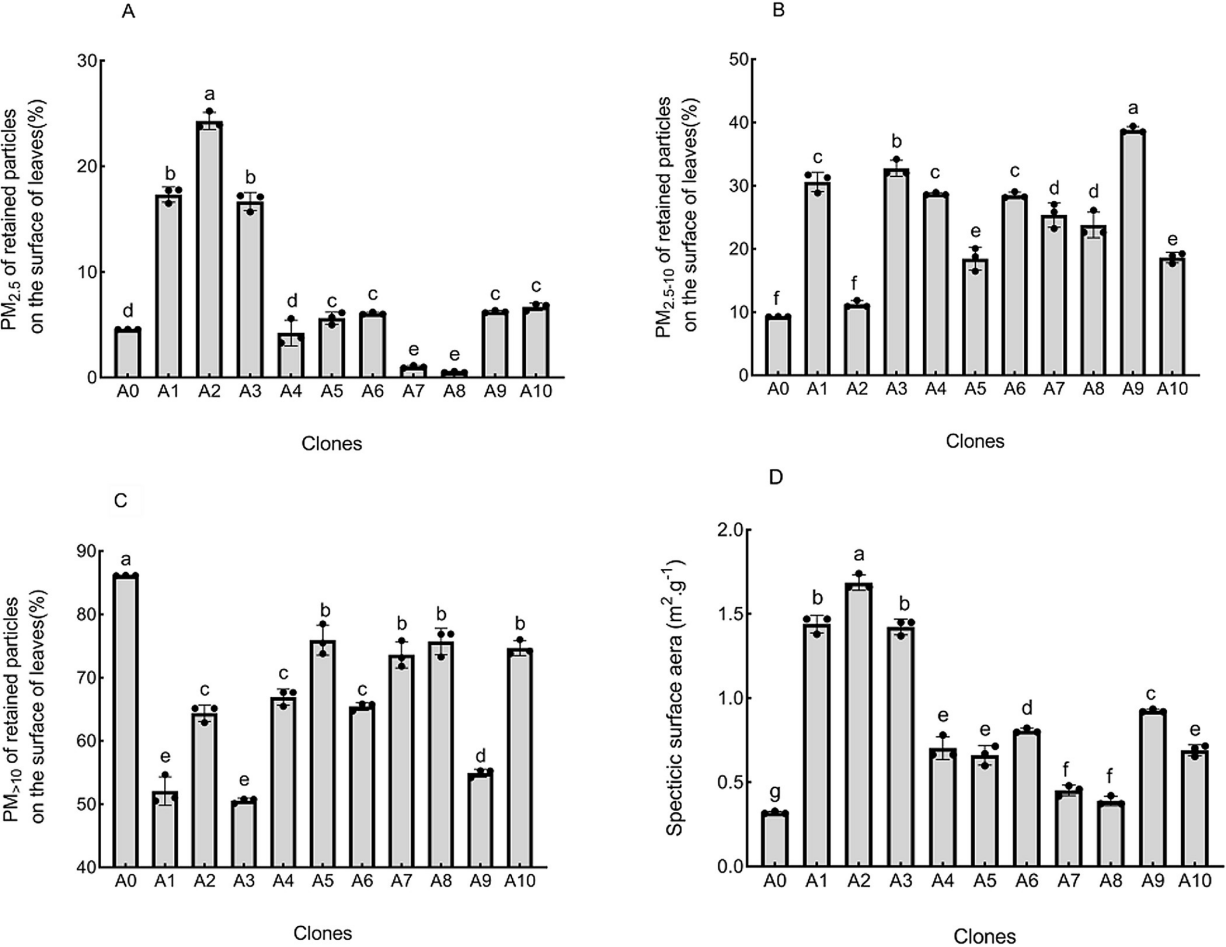
Particle size analysis for leaves of different clones of *Sophora japonica* L.

*3*.*1*.*2*.*1 Capacity of leaf surfaces to adsorb PM*_*2*.*5*._ PM_2.5_ may be inhaled into the bronchioles and alveoli, directly affecting lung function and increasing the susceptibility to hypoxia. The clones’ capacity to adsorb PM_2.5_ differed (Fig 2A), with an average COV of 6.14% indicating relative stability. A2 had the strongest PM_2.5_ retention capacity, 45.74–4498.16% greater than that of other clones. Multiple comparisons showed that A2 performed best and A1 and A3 performed well in the adsorption of atmospheric PM_2.5_ pollution; the performance of the other clones was relatively poor.

*3*.*1*.*2*.*2 Capacity of leaf surfaces to adsorb PM2*.*5–10*. PM_2.5–10_ is typically deposited in the upper respiratory tract and does not enter the alveoli upon inhalation; thus, its potential for harm is lower than that of PM_2.5_. The clones’ capacity to adsorb particles in this size class differed (Fig 2B), with an average COV of 3.59% indicating relative stability. The retention capacity of A9 was the strongest, 18.60–317.87% greater than that of other clones. Multiple comparisons showed that A9 performed best in adsorbing PM_2.5–10_ pollution, followed by A3. The other clones performed relatively poorly.

*3*.*1*.*2*.*3 Capacity of leaf surfaces to adsorb PM>10*. PM_>10_ is usually filtered in the nasal cavity and throat. Although they may cause discomfort, these particles do not enter the lungs; thus, their effects on human health are relatively minor. The clones’ ability to adsorb PM_>10_ particles differed (Fig 2C), with an average COV of 1.58% indicating relative stability. The surface retention capacity of A0 was the strongest, 13.45–65.45% greater than that of other clones. Multiple comparisons showed that A0 had the best retention capacity and that A5, A7, A8, and A10 performed well, whereas the other clones performed relatively poorly.

*3*.*1*.*2*.*4 Specific surface areas of retained particulate matter on leaf surfaces*. The specific surface area (SSA) is the total surface area per unit particle mass of a tested materials. Higher SSA values indicate more adsorption of harmful substances, and consequently greater environmental benefits. SSA differed among clones (Fig 2D), with an average COV of 3.86% indicating relative stability. A2 had the highest leaf surface SSA value, 17.13–427.63% higher than those of other clones. Multiple comparisons showed that A2 had the greatest environmental benefits. A1 and A3 also performed well, whereas the other clones performed relatively poorly.

#### 3.1.3 XRF analysis of particles retained on leaf surfaces

**Fig 3:**
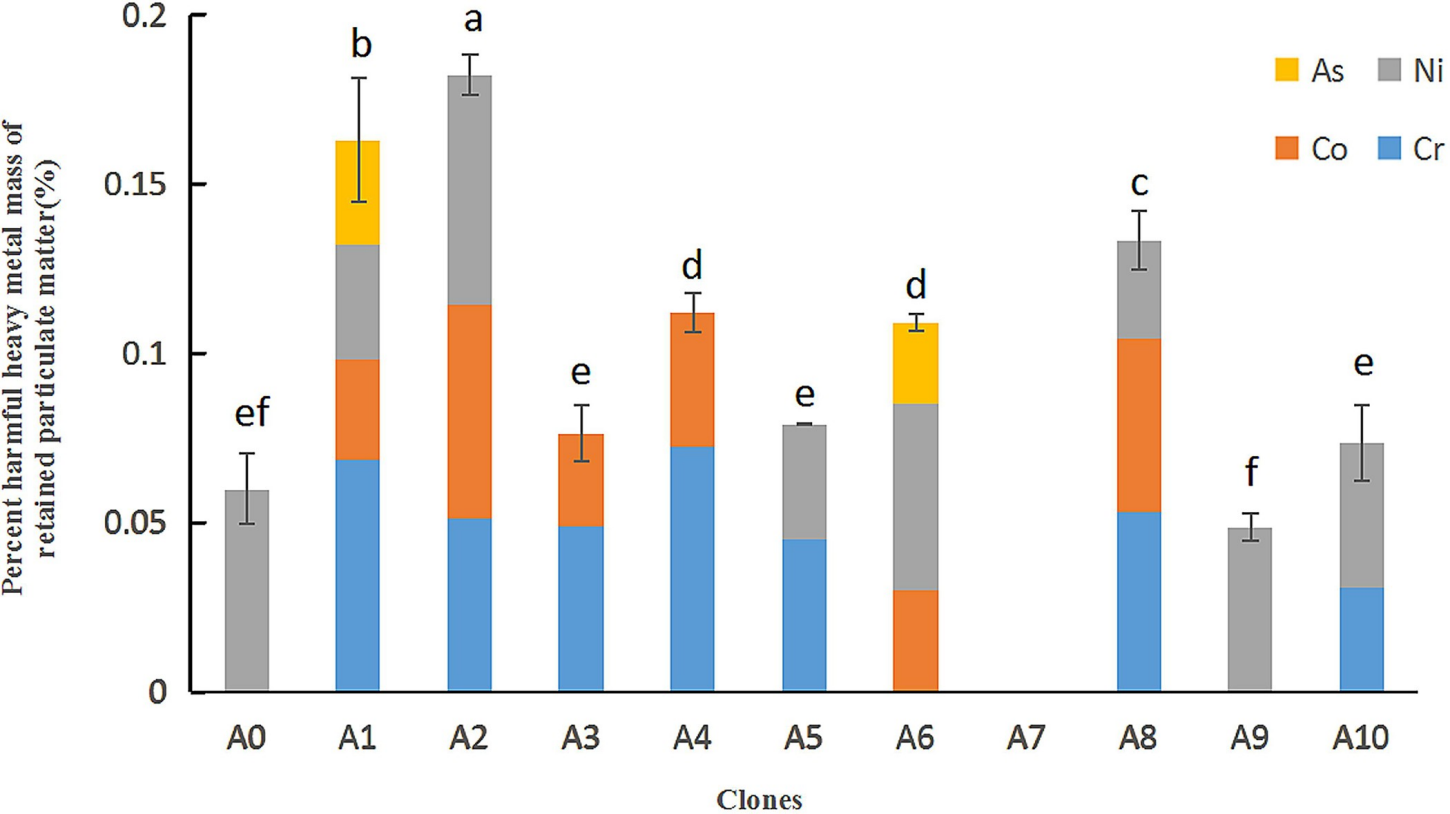
X-ray fluorescence (XRF) analysis of harmful heavy metals in particulate matter found on leaf surfaces.

We used XRF to identify and determine the mass ratios of heavy metals in the dust retained on leaf surfaces. Cobalt (Co), chromium (Cr), nickel (Ni), and arsenic (As) were detected in all clones except A7 (Fig 3).

Co, a silver-white ferromagnetic metal, may alter the blood serum protein composition and damage the lungs, pancreas, and other organs. A2 had the greatest Co retention, whereas no Co was detected in A0, A5, A7, A9, or A10. Cr is a highly toxic, silver-white metal that easily enters human cells and accumulates in tissues. It is carcinogenic and may induce gene mutation. A4 exhibited the strongest capacity to retain Cr, whereas no Cr was detected in A0, A6, A7, or A9. Ni is a nearly silvery-white, sensitizing heavy metal. Ni ions may penetrate the skin through pores or sebaceous glands, triggering hypersensitivity and inflammation. Once symptoms appear, Ni allergies may persist indefinitely. A2 had the greatest Ni retention, whereas no Ni was detected in A3, A4, or A7. As and many of its compounds have lethal toxicity and great potential to harm human health, and are often used in pesticides. As was detected only in clones A1 and A6, and A1 demonstrated the strongest retention capacity. The absence of As in other clones indicates poor As retention capacity.

A2 exhibited the greatest retention of heavy metals, at levels 11.85–211.99% greater than those of other samples. A2 had the best atmospheric heavy metal adsorption performance, A1 performed better than average, and the other clones performed relatively poorly, with A7 exhibiting the poorest adsorption capacity.

#### 3.1.4 Comprehensive dust retention capacity

**Fig 4:**
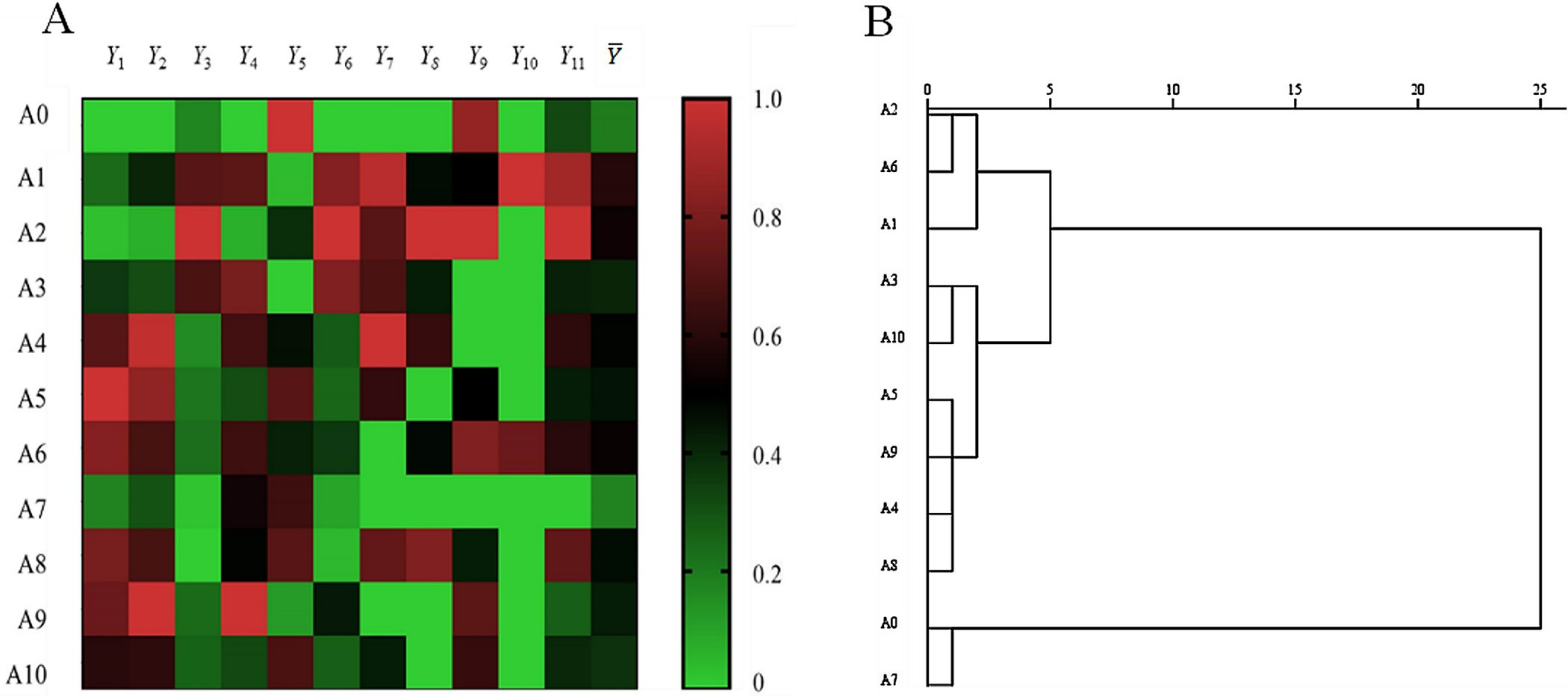
Thermal and cluster map of dust retention indexes (Y_1_–Y_11_). Data in 5A comprise the membership degree ($\bar{Y}$) of each sample, total natural and artificial particulate matter per unit leaf area, PM_2.5_, PM_2.5–10_, PM _> 10_, and specific surface area (SSA), and elemental content for chromium (Cr), cobalt (Co), nickel (Ni), and arsenic (As).

Because a single dust retention index cannot objectively and comprehensively reflect dust retention ability, we assessed the degree of membership of each index based on standardized data from 11 dust retention indices using the membership function method, and calculated the average membership degree of each clone. Larger values indicate stronger overall dust retention abilities. The order of the average comprehensive dust retention membership degrees among clones was as follows: A1 > A2 > A6 > A4 > A8 > A5 > A9 > A3 > A10 > A0 > A7 (Fig 4A). Further cluster analysis was conducted based on the average degree of membership of each sample (Fig 4B). The use of a minimum distance of 5 between groups yielded three groups based on comprehensive dust retention capacity. The first group consisted of clones A1, A2, and A6, which have relatively strong comprehensive dust retention capacities. The second group consisted of A4, A8, A5, A9, A3, and A10, with moderate comprehensive dust retention capacities, and the third group comprised A0 and A7, with relatively poor comprehensive dust retention capacities.

### 3.2 Leaf phenotypic traits

**Fig 5:**
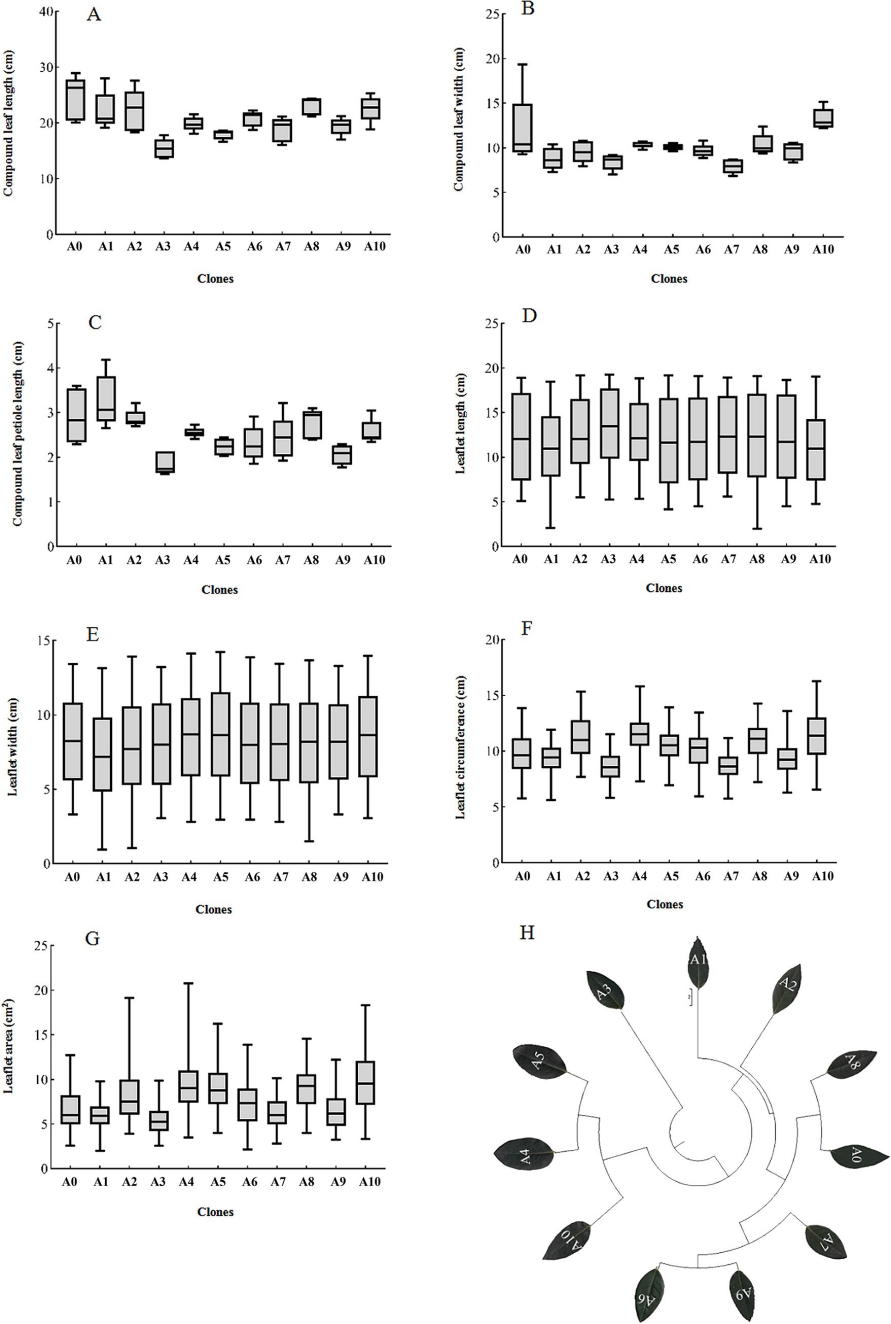
Unweighted pair group method with arithmetic mean (UPGMA) cluster analysis of the phenotypic traits of different clones of *Sophora japonica* L.

The phenotypic characteristics of the 11 clones are shown in Fig 5A–5G. A0 had significantly longer leaves than did the other clones, and leaf length did not differ among A1, A2, A8, and A10. A10 had the widest leaves, significantly wider than those of the other clones except A0. A1 had significantly longer petioles than did the other clones, with the exception of A3. Leaflet length did not differ significantly among clones. A4 had the widest leaflets, but this width differed significantly only from that of A1 leaflets. A10 had a significantly greater leaflet area than did the other clones. The results and change trend in leaflet perimeter were similar to those of leaflet area: A10 had the longest leaflet perimeter, differing significantly from that of the other clones except A2 and A4.

The clustering and distinction of clones using a single phenotypic trait index is difficult; we thus standardized nine phenotypic traits and clustered clones using the square Euclidean distance and average distance between groups. All samples were distinguished completely (Fig 5H). The use of the shortest distance between classes of 10 yielded three groups: class I comprised A3; class II comprised A4, A5, and A10; and class III comprised the remaining clones.

### 3.3 SEM and AFM analysis of leaf surfaces

#### 3.3.1 SEM characteristics

**Fig 6:**
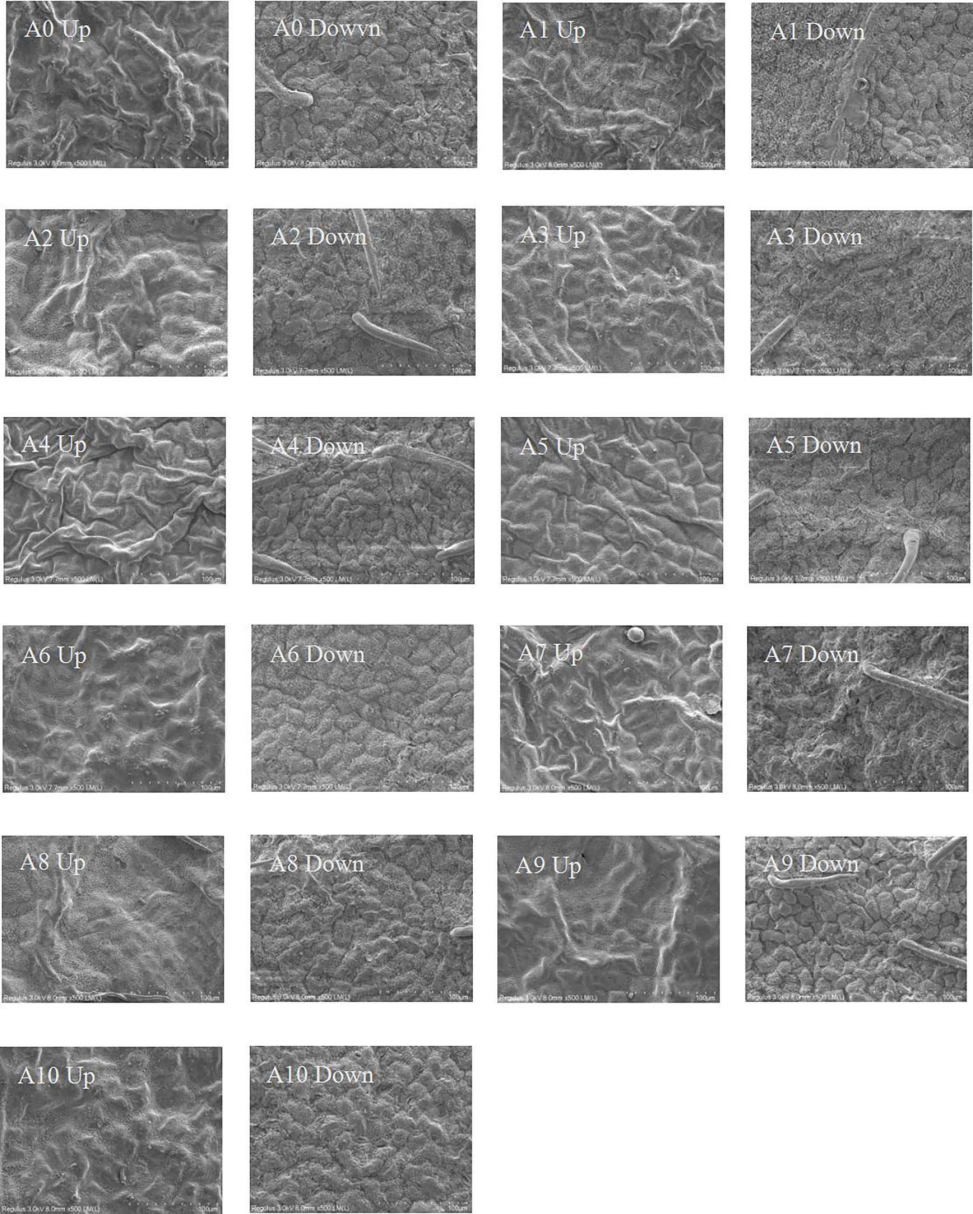
Scanning electron microscopy (SEM) of leaf epidermis micro-configurations in *Sophora japonica* L. (500×). Alphanumeric identifiers indicate the clone number and upper or lower epidermis of the leaf.

SEM analysis of the upper and lower leaf surfaces can be used to assess microscale differences in leaf surface characteristics among clones (Fig 6), based on the observation of the leaf surface micro-characteristics such as the number of gullies, stomatal apparatus morphology, the number of stomata and the number of epidermal hairs in different clones of *Sophora japonica*, and to convert SEM photographs into quantitative data (Table 2). The number of gullies on the upper and lower leaf surfaces differed among clones: A4 had the largest number of gullies and A8 had the fewest. All clones had similarly shaped, round and irregular stomata arrangement, but the number of stomata varied among clones, with A4 and A10 having the most stomata and A7 the fewest. Except for A6 and A9, stomatal size differed among clones. The degree of stomatal opening and closing also differed; the stomata of A3 were almost open, whereas the stomata of A4, A5, A7, A8, and A9 were nearly closed. A4 had the largest number of epidermal hairs, whereas no epidermal hair was observed on A1, A6, or A10 under 500× magnification. The roughness of the leaf surface also differed among clones, but these differences were not quantified.

**Table 2:** Characteristics of the epidermis and stomata (500×).

Clones	Number of gullies	Morphology of stomatal apparatus	Number of pores	Number of epidermal hairs
A0	86	Oblong, mostly closed, uneven in size, irregular in arrangement, about 80–150μm in diameter	23	1
A1	69	Oval, mostly closed, uneven in size, irregular in arrangement, about 100–120μm in diameter, a small number of guard cells protruding	17	0
A2	62	Oblong, mostly closed, uneven in size, irregular in arrangement, sunken, about 110–200μm in diameter	14	3
A3	60	Oval, mostly open, uneven in size, irregular in arrangement, sunken, about 100–120μm in diameter	19	2
A4	100	Oval, almost all closed, uneven in size, irregular in arrangement, sunken, about 40–100μm in diameter	31	5
A5	77	Oblong, almost all closed, uneven in size, irregular in arrangement, about 100–170μm in diameter	11	2
A6	79	Oval, small open, uniform size, irregular arrangement, sunken, about 100μm in diameter	17	0
A7	63	Oval, almost all closed, uneven size, irregular arrangement, sunken, diameter about 20–100μm	3	1
A8	53	Oblong, almost all closed, uneven size, irregular arrangement, sunken, about 40–80μm in diameter	15	4
A9	93	Oblong, almost all closed, uniform in size, irregular in arrangement, sunken, about 90–110μm in diameter	16	2
A10	65	Oval, small open, uneven size, irregular arrangement, sunken, about 150–180μm in diameter	33	0

Note: Numbers of surface hairs and gullies represent 500 times their sum on the upper and lower surfaces of the visual field of the scanning electron microscope (SEM).

#### 3.3.2 AFM characteristics

SEM and AFM can be used to assess nanoscale differences in leaf surface among clones, as it enables the accurate quantification of roughness and gully depth. Based on the comprehensive dust retention capacity results, we selected four clones for AFM testing: A1, which had the strongest dust retention capacity; A5, which had moderate retention capacity; A7, which had the worst dust retention capacity; and A0. To convert the three-dimensional images into quantitative data, we measured the contour arithmetic mean deviation (Ra) and peak-valley (P-V) of the measured parameters, which indicated the surface roughness and gully depth, respectively.

**Fig 7:**
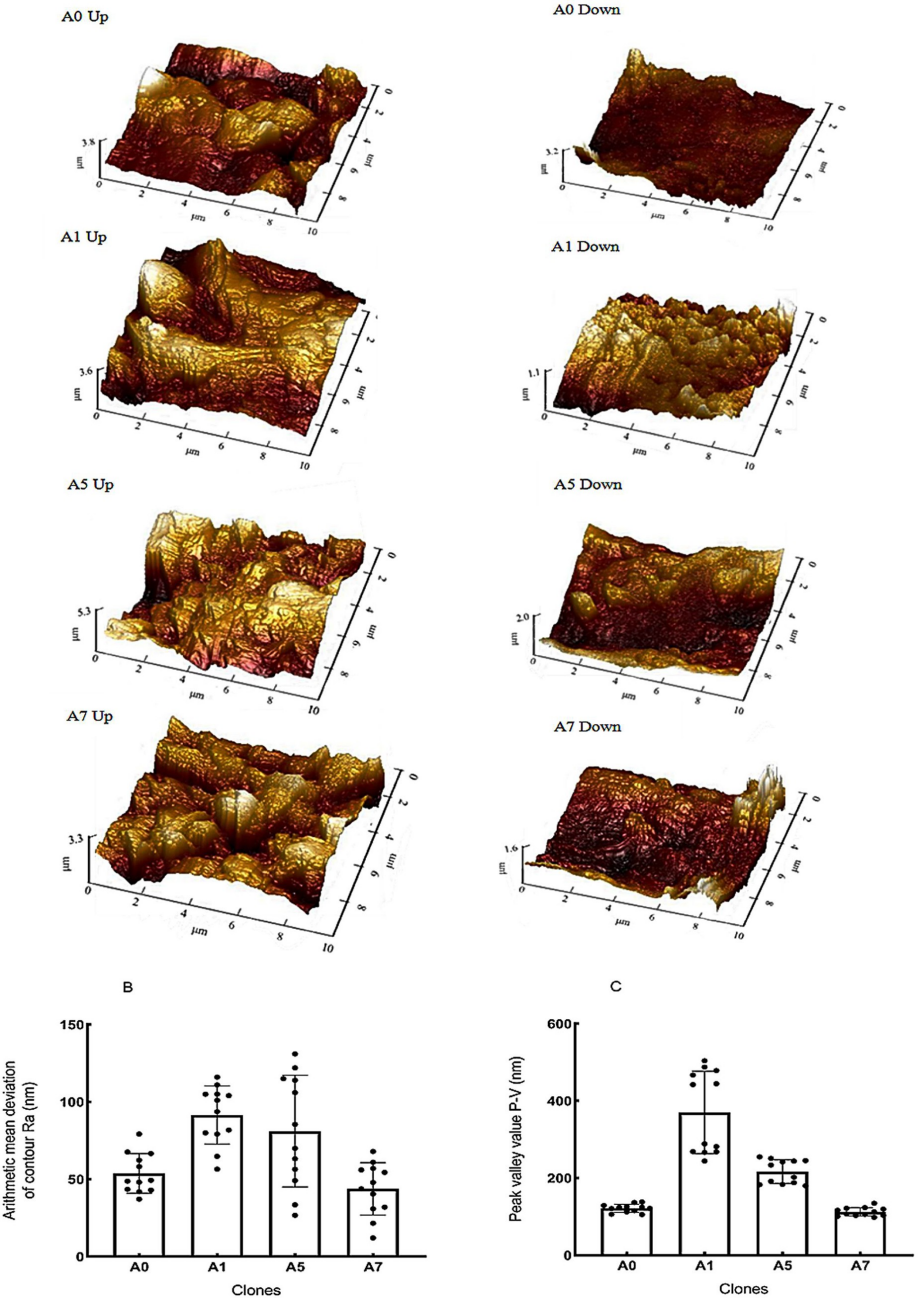
Microstructure of a three-dimensional leaf surface from each sample in atomic force microscope (AFM) view. Alphanumeric identifiers indicate the clone number and upper or lower epidermis of the leaf.

**Fig 8:**
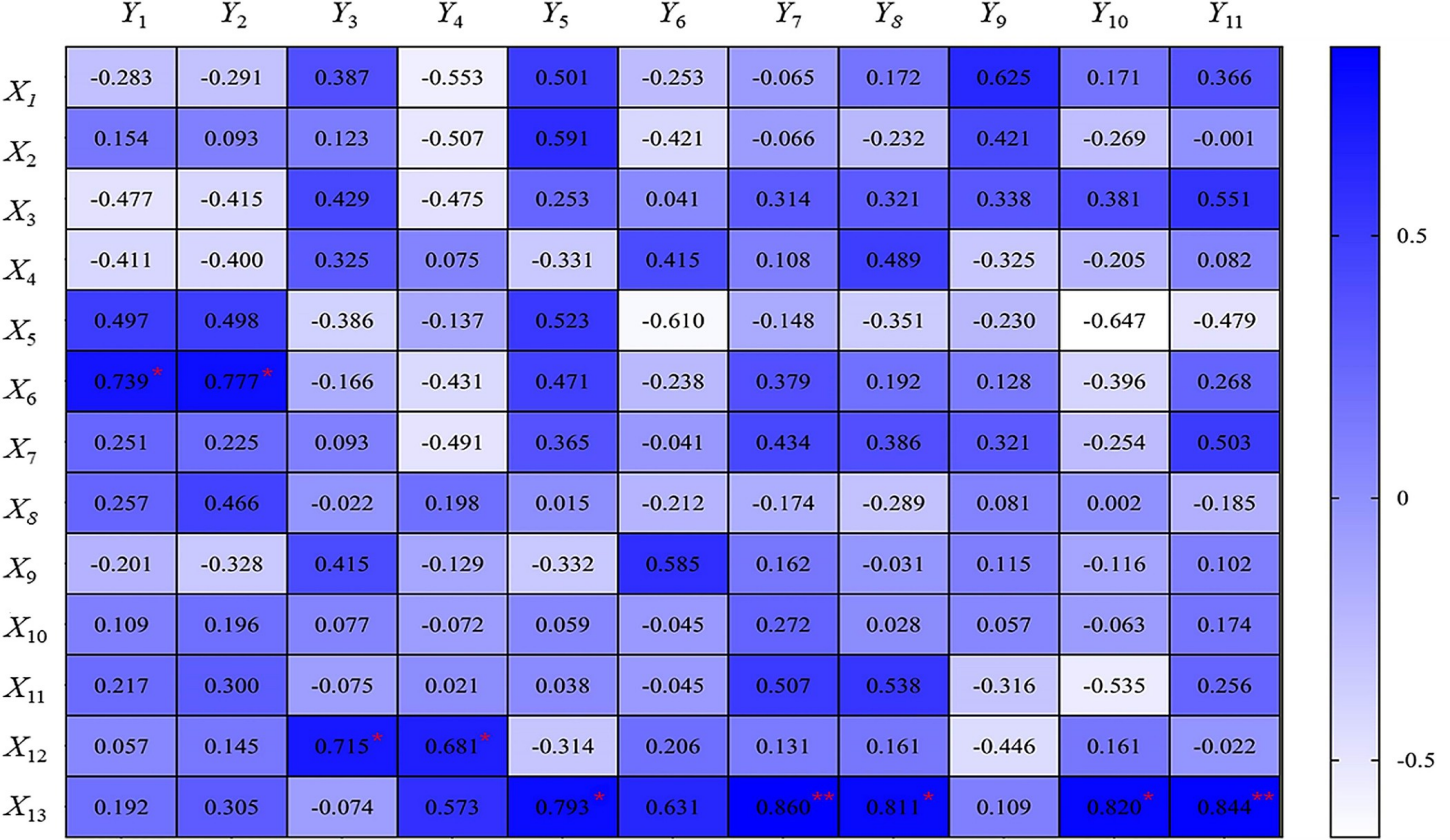
Relationship between leaf shape, SEM and AFM characteristics of the leaf surface, and dust retention index. Data are inter-index correlation coefficients (r). Significant correlations are indicated by *(*P* < 0.05) and ** (*P* < 0.01). Leaf shape and surface microtrait indicators include compound leaf length (X_1_), compound leaf width (X_2_), compound leaf petiole length (X_3_), leaflet length (X_4_), leaflet width (X_5_), leaflet area (X_6_), leaflet circumference (X_7_), number of gullies (X_8_), stomatal size (X_9_), number of stomata (X_10_), epidermal hair number (X_11_), roughness (X_12_), peak and valley (X_13_). Dust blocker indicators (*Y*_*1*_–*Y*_*11*_) are consistent with Fig 4A.

AFM photographs of the samples revealed significant differences (Fig 7). Ra and P-V values, reflecting gully depth, were in the order of A1 > A5 > A0 > A7 (Fig 8B and 8C), consistent with the comprehensive dust retention capacity results. Leaf surface roughness and gully depth affect the dust retention capacity of blades, and higher Ra and P-V values indicate greater comprehensive dust retention capacity.

### 3.4 Relationships of leaf morphology and microstructure to dust retention capacity

To further explore leaf surface dust retention mechanisms, we standardized raw data for leaf shape, the SEM and AFM index, and the dust retention index for correlation analysis, and identified leaf morphological and surface microscopic characteristic indices that were related significantly to the dust retention ability (Fig 8). The leaflet area and total dust retention per unit leaf area under natural and artificial conditions correlated positively (correlation coefficients = 0.739 and 0.777, respectively), indicating that larger leaflet areas were associated with greater retention capacity. Ra correlated positively with PM_2.5_ and PM_2.5–10_ (correlation coefficients = 0.715 and 0.681, respectively), indicating that greater roughness was associated with better retention of particles in these two size classes. P-V values correlated positively with PM_>10_ retention capacity and Co and As contents (correlation coefficients = 0.793, 0.811, and 0.820, respectively), and strongly with the total heavy metal and Cr contents (correlation coefficients = 0.844 and 0.860, respectively), indicating that gully depthon the leaf surface significantly affected the retention effect of each sample on heavy metal pollutants and PM_> 10_ particles, where greater gully depth indicates a better retention effect.

## 4. Discussion and conclusion

The environmental quality bulletins of Hebei Province from the past 5 years indicate that the ambient air quality in Baoding City has reached the national secondary standard less than 60% of the time, and that air pollution levels have consistently ranked among the top 10 in China. Although the number of days on which the standard is reached has increased over time, the air quality situation remains problematic. However, plant leaves have obvious retention effects for atmospheric particulate pollutants, and the dust retention capacity of different tree species may reach more than 40 times [28,29] the strategic selection of dust-retaining tree species may improve the air quality and quality of life.

We demonstrated that leaf shape differed among the tested clones, the reason may be due to the dual effects of cultivation environment and genetic variation [30]. The artificial dust retention treatment was comparable to dust retention under natural conditions, confirming the arguments presented by Guo *et al*. [10]. The average total particulate matter retention per unit leaf area per clone was 85.05 μg·cm^2^, which was greater than that of common ornamental tree species used in northern China, including *Euonymus japonicus*, *Fraxinus chinensis*, and *Acer truncatum* [31]. This finding implies that the overall dust retention capacity of *S*. *japonica* is better than that of these other tree species, potentially due to interspecific differences in leaf surface microstructure. With the exception of A6, the leaf surfaces of the tested clones had epidermal hairs, which increase the retention of particles per unit leaf area; this observation may be related to the air quality conditions at the time of sampling and the sampling location. We analyzed total particle size using a laser particle size analyzer. Most particles in the samples were in the PM_<10_ size class, and retention capacity differed among size classes. These results are consistent with those of Wang et al. [32] and Tomaevi et al. [33] reported that most particles retained on leaf surfaces were 10 μm in size. Conversely, Zhang et al. [31], who reported that most particles retained by six plants in Beijing were in the range of 10–50 μm, this difference may be attributable to differences among tree species or in the timing of sampling. We also analyzed heavy metal content in the particles retained on the leaf surfaces using XRF, and detected Co, Cr, Ni, and As. The average percentage of total heavy metals was 0.09%, and the percentage of total harmful heavy metals in A2 was the highest, which was lower than that observed on weeping willow, elm, and other common tree species in northern China [34] but differences may be a result of the use of different testing methods: XRF is used to identify all particles for element analysis, whereas the X-ray energy spectrum method is used to determine the elemental compositions of single particles. The two methods each have advantages and disadvantages. X-ray spectrometry is more efficient in terms of time and labor, whereas XRF technology is more accurate and objective, but also costlier. In this study, SEM & AFM techniques were used to observe the leaf surface microscopic characteristics of each sample. The analysis showed that there were significant differences in leaf surface microstructure of most of the tested clones, and the correlation analysis showed that the ability of retaining atmospheric particles of different clones mainly depended on the differences in leaf surface microstructure. The number and depth of leaf furrows, the size and number of stomata, the roughness of leaf surface and the number of epidermal hairs were the main reasons for the differences in dust-retention ability among the clones. The reason might be that leaf furrows increased leaf surface roughness, and stomata and fur increased the contact area between leaf surface and air. The enhanced dust-catching ability of leaf surface [35–37] may also be due to the fact that atmospheric particles contact with the surface fur and enter through the cracks in the leaf’s cuticle, thus increasing the dust-catching ability of leaf surface as a whole [38,39]. The SEM & AFM index, and the dust retention index revealed a significant positive correlation between the leaflet area and total dust retention per unit leaf area under natural and artificial conditions. Ra correlated positively with the capacity to retain PM_2.5_ and PM_2.5–10_, and P-V values correlated positively with the capacity to retain PM_10_, Co, and As. We observed a highly significant positive correlation between the heavy metal and Cr contents. Differences in dust retention capacity among clones may be explained by microscopic differences in leaf surface characteristics. Our results align with those of other studies, including Zhang *et al*. [26] and Lu *et al*. [14] but contradict those of Sun *et al*. [40] that leaf surface gullies are unrelated to the dust retention capacity of six ornamental plants in Kunming. Gullies may facilitate the adsorption of fine particles on inner gully walls; thus, these conflicting results may also reflect differences in climatic conditions or tree species between locations. The conclusion of this study also differs from the finding of Gao *et al*. [41] that stomatal Pb accumulation in Chinese cabbage leaves was proportional to the effects of stomatal retention and absorption of PM_2.5_, which may be species dependent.

We demonstrated that dust retention testing under artificial conditions yields results comparable to those of tests conducted under natural conditions, and may be used as an alternative in the case of uncontrollable external factors, such as extreme weather. *S*. *japonica* exhibited excellent dust retention capacity, with significant differences observed among clones. A5 had the strongest overall retention capacity. A2 had the strongest capacity to retain PM_2.5_, A9 had the strongest capacity to retain PM_2.5–10_, A0 had the strongest capacity to retain PM_>10_, and A2 had the highest SSA value and greatest capacity to adsorb heavy metals. Overall, the comprehensive dust retention capacity of A1 was the strongest, that of A5 was moderate, and that of A7 was poorest. The dust retention capacity should be considered when breeding new ornamental tree varieties, and new varieties that exhibit both ornamental value and anti-pollution functions should be bred. We also demonstrated that differences in dust retention capacity among clones can be explained effectively by leaf morphology and the SEM and AFM index, thereby providing a new tool for future studies.

## Supporting information

S1 Fig Retention capacity of total particulate matter in leaves of different clones of *Sophora japonica* L. Data in 2A are means ± standard deviation. Different lowercase letters indicate significant differences among different clones using the same determination method (*p* < 0.05).
(XLSX)

S2 Fig Particle size analysis for leaves of different clones of *Sophora japonica* L. (XLSX)

S3 Fig X-ray fluorescence (XRF) analysis of harmful heavy metals in particulate matter found on leaf surfaces. (XLSX)

S4 FigThermal and cluster map of dust retention indexes (Y_1_–Y_11_).Data in 5A comprise the membership degree ($\bar{Y}$) of each sample, total natural and artificial particulate matter per unit leaf area, PM_2.5_, PM_2.5–10_, PM _> 10_, and specific surface area (SSA), and elemental content for chromium (Cr), cobalt (Co), nickel (Ni), and arsenic (As).(XLSX)

S5 Fig Unweighted pair group method with arithmetic mean (UPGMA) cluster analysis of the phenotypic traits of different clones of *Sophora japonica* L. (XLSX)

S6 Fig Scanning electron microscopy (SEM) of leaf epidermis micro-configurations in Sophora japonica L. (500×). Alphanumeric identifiers indicate the clone number and upper or lower epidermis of the leaf.
(XLSX)

S7 Fig Microstructure of a three-dimensional leaf surface from each sample in atomic force microscope (AFM) view. Alphanumeric identifiers indicate the clone number and upper or lower epidermis of the leaf.
(XLSX)

S8 Fig Relationship between leaf shape, SEM and AFM characteristics of the leaf surface, and dust retention index. Data are inter-index correlation coefficients (r). Significant correlations are indicated by *(*P* < 0.05) and ** (*P* < 0.01). Leaf shape and surface microtrait indicators include compound leaf length (X_1_), compound leaf width (X_2_), compound leaf petiole length (X_3_), leaflet length (X_4_), leaflet width (X_5_), leaflet area (X_6_), leaflet circumference (X_7_), number of gullies (X_8_), stomatal size (X_9_), number of stomata (X_10_), epidermal hair number (X_11_), roughness (X_12_), peak and valley (X_13_). Dust blocker indicators (*Y*_*1*_–*Y*_*11*_) are consistent with Fig 4A.
(XLSX)

S1 Table Clones tested. (XLSX)
